# Baseline characteristics for INVOKE‐2: a phase 2 randomized, double‐blind, placebo‐controlled study evaluating AL002 in early Alzheimer’s disease

**DOI:** 10.1002/alz.095594

**Published:** 2025-01-09

**Authors:** Arthur J Mayorga, Brady Burgess, Tuan Nguyen, Jingjing Gao, Giacomo Salvadore, Gary Romano

**Affiliations:** ^1^ Alector, South San Francisco, CA USA

## Abstract

**Background:**

AL002 is a humanized, TREM2‐selective, agonistic IgG1 monoclonal antibody. The INVOKE‐2 study is the first to test the efficacy and safety of a TREM2 agonistic antibody in participants with Alzheimer’s Disease (AD). A Phase 1 study of AL002 demonstrated dose‐dependent target engagement and dose‐dependent effects on microglial signaling biomarkers; AL002 was well tolerated at all doses tested.

**Method:**

The INVOKE‐2 study is a randomized, double‐blind, placebo‐controlled 4‐arm, common close study (48‐96 weeks of treatment) with a dose‐blind 48‐week long‐term extension. In addition to the assessment of clinical efficacy and safety, the study includes biomarkers of target engagement, microglial signaling, and AD pathophysiology (including colony‐stimulating factor 1 receptor [CSF‐1R], osteopontin, interleukin‐1 receptor antagonist [IL1RN], phosphorylated tau217 [ptau217], microtubule binding region of tau‐243 [MTBR‐tau243], phosphorylated tau181 [ptau181], amyloid‐beta 42/40, amyloid positron emission tomography [PET], tau PET, glial fibrillary acidic protein [GFAP], neurofilament light chain [NfL], neurogranin, YKL‐40, and volumetric MRI).

**Result:**

A total of 381 participants were randomized in INVOKE‐2. The median age of participants was 71 years (range: 51‐85 years), with 78% of participants 65 years of age or older. Overall, 50% of participants were female and 94% were Caucasian. The clinical diagnosis at enrollment was mild cognitive impairment due to AD for 67% of participants and mild dementia due to AD for 33% of participants. Treatment‐emergent brain MRI changes resembling amyloid‐related imaging abnormalities have been observed in the study. These changes were of greater incidence and severity in homozygous ApoE4 carriers, leading to the discontinuation of homozygous ApoE4 carriers early in the study. Of the 381 participants, 59% were heterozygous ApoE4 carriers. Clinical outcome assessment data at baseline were consistent with the Food and Drug Administration (FDA) definition of an early AD population. Amyloid positivity was confirmed in all participants prior to enrollment by analysis of cerebrospinal fluid or amyloid PET. For those participants (n = 242) with amyloid PET assessed at baseline, the mean (standard deviation [SD]) in centiloids was 100.4 (38.7).

**Conclusion:**

Taken together, the baseline characteristics data for the INVOKE‐2 study represent a study population that enables testing of the effects of a TREM2 agonist in early AD.

